# In-Wheel Motor Control System for Four-Wheel Drive Electric Vehicle Based on CR-GWO-PID Control

**DOI:** 10.3390/s23198311

**Published:** 2023-10-08

**Authors:** Xiaoguang Xu, Miao Wang, Ping Xiao, Jiale Ding, Xiaoyu Zhang

**Affiliations:** 1 School of Electrical Engineering, Anhui Polytechnic University, Wuhu 241000, China; xuxg@ahpu.edu.cn (X.X.); 2220320121@stu.ahpu.edu.cn (M.W.); 2220320123@stu.ahpu.edu.cn (J.D.); 2 School of Engineering, University of Bridgeport, Bridgeport, CT 06604, USA; 3 Anhui Province Key Laboratory of Intelligent Car Wire-Controlled Chassis System, Anhui Polytechnic University, Wuhu 241000, China; 2220110171@stu.ahpu.edu.cn

**Keywords:** micro-electric vehicle, in-wheel motors, Chaotic Random grey wolf control algorithm, speed control

## Abstract

In order to improve the driving performance of four-wheel drive electric vehicles and realize precise control of their speed, a Chaotic Random Grey Wolf Optimization-based PID in-wheel motor control algorithm is proposed in this paper. Based on an analysis of the structural principles of electric vehicles, mathematical and simulation models for the whole vehicle are established. In order to improve the control performance of the hub motor, the traditional Grey Wolf Optimization algorithm is improved. In particular, an enhanced population initialization strategy integrating sine and cosine random distribution factors into a Kent chaotic map is proposed, the weight factor of the algorithm is improved using a sine-based non-linear decreasing strategy, and the population position is improved using the random proportional movement strategy. These strategies effectively enhance the global optimization ability, convergence speed, and optimization accuracy of the traditional Grey Wolf Optimization algorithm. On this basis, the CR-GWO-PID control algorithm is established. Then, the software and hardware of an in-wheel motor controller are designed and an in-wheel motor bench test system is built. The simulation and bench test results demonstrate the significantly improved response speed and control accuracy of the proposed in-wheel motor control system.

## 1. Introduction

With the ability to cater to daily commuting needs, micro-electric vehicles designed for urban mobility have gained significant public favor. When compared to the conventional propulsion systems of electric vehicles, the approach utilizing four in-wheel motors to drive the electric vehicle stands out [1]. This design—which integrates the motor with the wheel—allows for direct motor-driven wheel movement, thereby negating the need for components such as the engine, clutch, main reducer, and differential. However, achieving widespread adoption of in-wheel motors in the automotive industry still requires more in-depth research at present. Due to current limitations in material development, in-wheel motors still face issues related to quality and durability. However, with continuous technological advancements, new materials capable of withstanding demanding road conditions and environmental factors are emerging, providing fundamental solutions to these issues. Therefore, considering our understanding of development future trends, the prospect of using four-wheel in-wheel motors to drive micro-electric vehicles holds immense potential and warrants comprehensive exploration. The distinctive merits of such a configuration, including compact structure, high power transmission efficiency, and exceptional electric control performance, have garnered substantial attention in both domestic and international automotive industries. Given these combined advantages, the prospect of employing a four-wheel in-wheel motor to drive micro-electric vehicles holds significant potential and is deserving of comprehensive exploration. For example, Vishnu et al. [2] proposed a brushless DC in-wheel motor drive mode in 2020 and developed hardware equipment for practical applications, providing a new drive mode for electric vehicles. In addition, Li et al. [3] have proposed multi-modal driving force distribution strategies to improve the reliability and independence of four-wheel in-wheel drive electric vehicles. Their results revealed that such an approach can meet the expected requirements. Subroto et al. [4] contend that one of the key challenges in Four-Wheel Independent Drive Electric Vehicles (4WID EV) is how to allocate torque to each wheel effectively to improve the vehicle’s dynamic stability. To address this issue, scholars have designed a novel sliding mode controller with an adaptive proportional-integral (PI) sliding surface for more precise control. Similarly, Leng et al. [5] proposed a torque control method for a Four-Wheel Drive Electric Vehicle based on an allocation algorithm, and the stability of in-wheel motors were enhanced. The torque distribution strategy of a four-wheel independent-drive electric vehicle with optimal energy consumption proposed by Guo et al. [6] pays more attention to saving energy on the basis of improving the vehicle’s performance. In 2021, Silva et al. [7] proposed an optimized fuzzy logic control for application to four-wheel independent-drive electric vehicles. Through cooperative calculation in a genetic algorithm, the optimal combination of steering angle and sideslip angle can be found, thus reducing the processing time and error. When Jeong et al. [8] studied the automatic driving of electric vehicles, they proposed a tracking control strategy to drive four-wheel in-wheel motors. Dhamija et al. [9] have developed a torque vector control scheme for the in-wheel motor of four-wheel drive electric vehicles based on non-linear model predictive control. Later, Saleeb et al. [10] developed a driving strategy based on an artificial neural network for direct torque control to drive electric vehicles, which reduced the core loss and improved the driving efficiency.

Indeed, an examination of the domestic and international literature reveals that scholars have put forward an array of strategies and control approaches for the four-wheel in-wheel motor drive. However, there remains a dearth of studies focused on motor speed control to enhance the overall dynamic performance of vehicles. A well-designed control scheme has the potential to increase the vehicle’s driving performance while simultaneously curbing energy consumption. This underscores the necessity of further research in this particular direction.

To maintain the reasonable acceleration and driving speed of electric vehicles, there have also been relevant studies using PID control. Rohan A. [11] proposed a strategy based on fuzzy PID control to improve the response performance of the PID controller. Savnani et al. [12], after modeling a four-wheel drive vehicle, proposed that a continuous PID can be used to control the error, which is the best control strategy. Through calculations, the drive can be controlled within a short time to achieve the best motor control effect. Traditional PID control has the problem of inaccurate adjustment parameters, which affect the accuracy of the PID controller. In 2021, Altbawi et al. [13] designed a fractional order PID controller based on a gradient optimization algorithm, which has better control characteristics than the traditional PID controller. Muqeet et al. [14] also proposed a PID controller based on the meta-heuristic optimization algorithm. The test results indicated that the robustness and stability were improved. Later, Zhang et al. [15] applied adaptive PID control to the speed control of an in-wheel motor. Due to the reliability, universal applicability, and maturity of PID control, this study proposes a PID controller based on an optimization algorithm, applying the improved PID controller to a four-wheel-drive electric vehicle driven by in-wheel motors.

In order to solve the shortcomings of poor robustness and slow convergence inherent to the PID controller, Wang et al. [16] proposed a modified firefly swarm optimization algorithm based on the original firefly swarm optimization algorithm. Their results showed that the performance of the controlled brushless DC motor was improved, thus illustrating that the group optimization algorithm has a good effect on the optimization of the PID controller. In addition, Mittal [17] proposed the modified grey wolf optimizer (MGWO) algorithm, which is used for the image segmentation calculation problem. In order to obtain the optimal solution, they showed the universality of the application of the optimization algorithm. Mirjalili et al. [18] proposed a meta-heuristic Grey Wolf Optimization (GWO) algorithm inspired by the collective behavior of wolves. However, the GWO algorithm has the disadvantages of premature convergence and poor global search ability. In view of these shortcomings, different researchers have proposed various strategies. In 2022, Biabani et al. [19] developed a new optimization algorithm combining the gravity search algorithm, particle swarm optimization algorithm, and Gray Wolf optimization algorithm (HGPG) is developed. The HGPG algorithm provides significant improvements, in terms of exploration and development. Duan et al. [20] have also detailed the advantages of hybrid algorithms in terms of improving the performance of GWO and developed a new algorithm called GWO-SCA. The exploration ability of the sine cosine algorithm (SCA) was used to optimize the Grey Wolf Optimization algorithm, in order to make up for its weak global search ability. This strategy also inspired a new idea for the optimization of the GWO algorithm, as detailed in this paper.

In the realm of engineering applications, Mahdis et al. [21] harnessed Grey Wolf Optimization to address engineering challenges. Concurrently, researchers such as Precup [22] have proposed robot path-planning approaches based on the grey wolf optimizer. Addressing specific scenarios, scholars such as Wen [23] have developed grey wolf optimizers augmented by random opposition learning. Dutta [24] and Nayak [24] have also presented enhanced methods for optimizing PID controllers using the GWO algorithm. However, these approaches have not yet addressed the limitations of the GWO algorithm. Given the potential for enhancing the GWO optimization algorithm and the limited exploration of its application in optimizing motor PID parameters within the new energy engineering context, this paper introduces a Chaotic Random Grey Wolf Optimization-based PID (CR-GWO-PID) control algorithm. The proposed algorithm aims to improve the overall operability, safety, and reliability of vehicles through the optimization of motor speed control.

## 2. Modeling and Simulation of Four-Wheel In-Wheel Motor Drive Electric Vehicles

### 2.1. Structure and Principle of Four-Wheel Drive In-Wheel Motor Electric Vehicles

**Figure 1:**
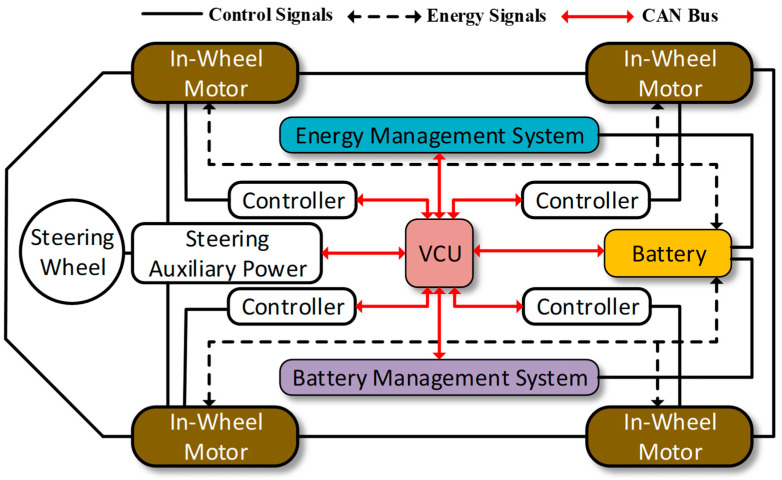
Structural diagram of a four-wheel drive in-wheel motor electric vehicle.

The in-wheel motor drive is utilized to directly integrate the motor assembly with the reducer in the in-wheel, and all four wheels of a vehicle can be directly driven by four brushless DC motors. Compared with traditional electric vehicles, the use of an in-wheel motor drive eliminates the differential, half-shaft, and even secondary transmission device. The advantages of simple mechanical structure, environmental protection, and high transmission efficiency have led in-wheel motors to be considered in the industry as the final drive form of electric vehicles [1]. As shown in Figure 1, when the stator and rotor move while energized, the electronic commutator (switching circuit) controls the sequence and time of stator winding energization, according to the position sensor signal, and generates a rotating magnetic field to drive the rotor to rotate. The electronic control unit (ECU) provides the controller that outputs the control signal to each motor through the CAN bus. The controller provides the motor commutator position signal according to the position sensor inside the motor, such that the motor can continue to run, reach the target speed, and drive stably. At the same time, the battery management system monitors and manages the battery pack in real-time, while the energy management system distributes electrical energy to each motor in real-time.

### 2.2. Vehicle Modeling

**Dynamics model.** According to longitudinal force analysis of the vehicle during driving and the resistance that the vehicle must overcome on the road [25], the driving equation of the vehicle is as follows:

(1) $$F_{t}=F_{f}+F_{w}+F_{i}+F_{j},$$

where *F_t_* is the driving force, *F_f_* is the rolling resistance, *F_w_* is the air resistance, *F_i_* is the slope resistance, and *F_j_* is the acceleration resistance.

(2) $$F_{f}=f\;m\;g,$$

where *f* is the rolling resistance coefficient, *m* is the vehicle’s mass and g is gravitational acceleration. The rolling resistance coefficient is affected by road conditions and vehicle speed changes.

(3) $$F_{w}=\frac{\rho C_{D}Av_{a}^{2}}{2},$$

where *ρ* is the air density, *C_D_* is the air resistance coefficient, *A* is the windward area of the vehicle, and *F_w_* is calculated according to the speed of the vehicle *v_a_* (m/s).

(4) $$F_{i}=mg\sin\alpha ,$$

where *α* is the slope of the ramp.

(5) $$F_{j}=\delta m\frac{dv_{a}}{dt},$$

where *δ* is the mass–ratio coefficient. The motor and the in-wheel are rigidly connected, and the in-wheel directly obtains the driving force, *F_t_*, as shown in Equation (6):

(6) $$F_{t}=fmg+\frac{\rho C_{D}Av_{a}^{2}}{2}+mg\sin\alpha +\delta m\frac{dv_{a}}{dt}.$$

**Battery model**. The battery can be seen as an ideal voltage source and a resistor in series.

(7) $$E_{0}=U+Ir,$$

where *U* is the working voltage, *I* is the working current, and *r* is the internal resistance of the battery. The discharge power of the battery is:

(8) $$P_{b}={\left(\frac{E_{0}}{R+r}\right)}^{2}R,$$

where *R* is load resistance Then, a new variable *x* is introduced, indicating the ratio of the internal resistance to the load resistance. The discharge power equation of the battery is replaced by:

(9) $$P_{b}={\left(\frac{E_{0}}{1+x}\right)}^{2}R.$$

The actual maximum output power can be calculated by Equation (10):

(10) $$P_{bMAX}=\frac{{E_{0}}^{2}}{4R},$$

where *P_bMAX_* is the maximum output power of a single cell. When *x* = 1, that is, *R* = *r*, the maximum power is output. The parameters of the battery depend on the target mileage of the car and the maximum power of the motor. The number of battery packs can be determined based on the maximum power of the motor:

(11) $$n_{1}=\frac{P_{eMAX}}{P_{bMAX}\eta_{e}\eta_{ec}},$$

where *P_eMAX_* is the maximum power of the motor; *η_e_* and *η_ec_* are the working efficiency of the motor and the efficiency of the motor controller respectively. Through calculation, *n*_1_ ≈ 120, and they are connected in series to achieve the maximum operating voltage for the vehicle’s in-wheel motor. The number of batteries required to meet the vehicle’s target driving mileage (150 km) is:

(12) $$n_{2}=\frac{1000LW}{c_{b}v_{b}},$$

where *L* is the target cruising range, *W* is the energy consumed when the car travels 1 km, *c_b_* is the capacity of a single lithium battery, and *v_b_* is the voltage of a single lithium battery. The calculation shows that *n*_2_ ≈ 615, and these should be connected in parallel to achieve the target range of 150 km for the vehicle.

(13) $$N=SUM(n_{1},n_{2}).$$

Therefore, the final number of batteries in the battery pack is *N* = 735. The state of the battery pack during vehicle operation and charging is:

(14) $$SOC=SOC_{0}-\frac{\int_{0}^{t}Idt}{C_{n}}.$$

where *SOC*_0_ is the battery’s initial *SOC*, *I* is the working current, and *C_n_* is the battery rating.

**Motor model.** In this paper, a four-wheel in-wheel motor-driven urban mini-electric vehicle is studied. The motor power is 8 kW, 25.6 N·m. The relationship between vehicle speed and motor speed can be expressed by Equation (15). By simulating the road conditions and knowing the vehicle’s maximum driving speed *v_a_*, we can calculate the maximum speed of the motor *n_max_*.

(15) $$n_{max}=\frac{v_{a}}{0.377r},$$

**Figure 2:**
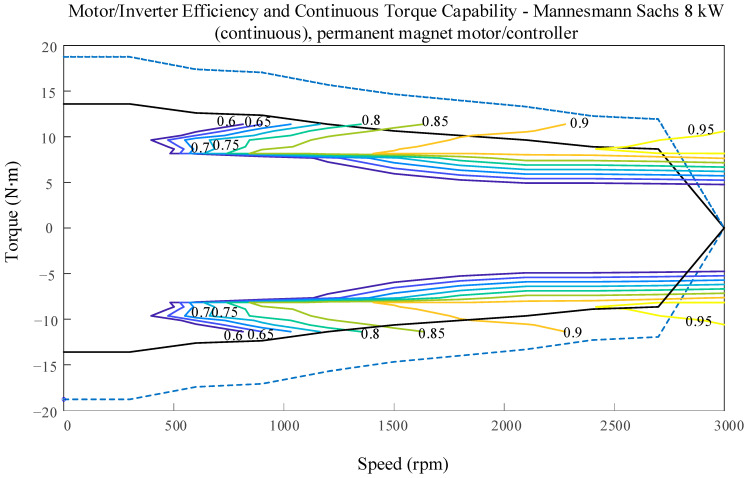
In-wheel motor map.

The maximum power of the motor can be calculated by Equations (16), where *a* is the vehicle’s acceleration. The in-wheel motor map, generated based on motor speed, torque, and efficiency, is depicted in Figure 2.

(16) $$P_{eMAX}=\frac{v_{a}}{3600\eta_{e}}\left(mgf+\frac{\rho C_{D}Av_{a}^{2}}{2}+\delta ma\right).$$

### 2.3. Vehicle Model Simulation and Analysis

**Figure 3:**
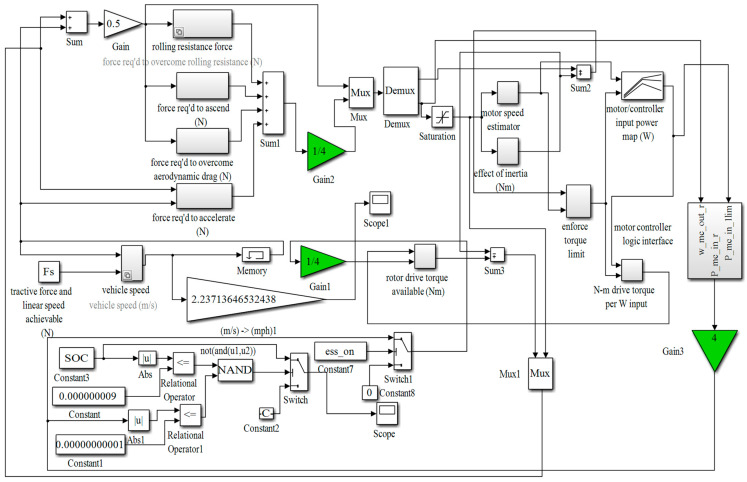
Vehicle model simulation diagram. (Green triangles indicate the changes in the number of in-wheel motors).

**Figure 4:**
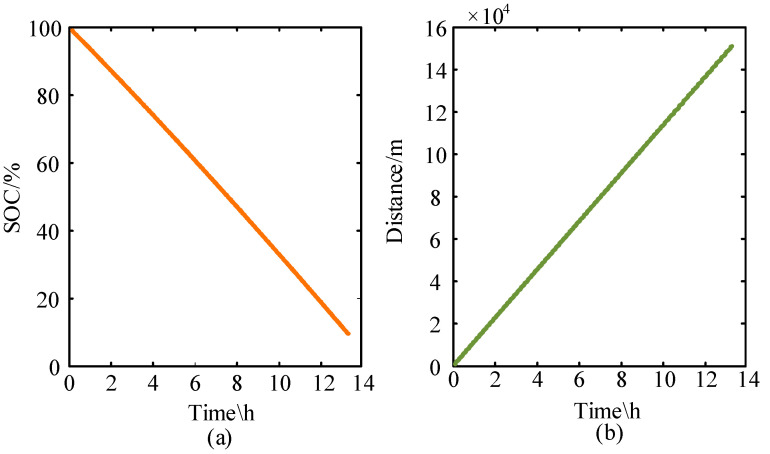
The results of vehicle driving simulation: (**a**) EV battery change and (**b**) EV mileage.

**Figure 5:**
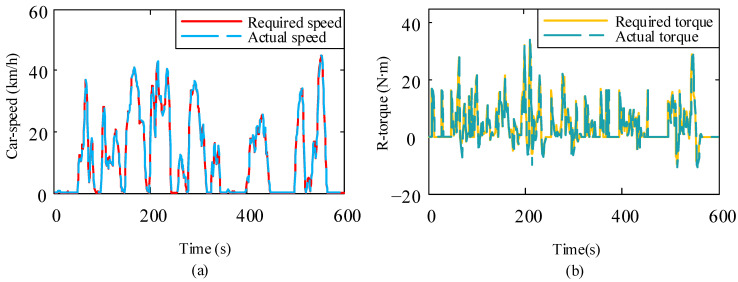
Comparison of key indicators of simulated driving under vehicle simulation road conditions: (**a**) Comparing the required EV driving speed with the actual EV driving speed; (**b**) Comparing the required motor torque of the EV with the actual motor torque of the EV.

For the designed simulation model of the micro-electric vehicle driven by a four-wheel in-wheel motor, the battery capacity is approximately 30.26 kWh. the motor power is 8 kW, and the vehicle parameters are provided in Table 1. Figure 3 shows the vehicle simulation model, incorporating kinematics, motor, and battery models. By simulating this vehicle model, we can derive the road condition requirements for the motor. The simulation model was tested under the road conditions of New York City (CYC_NYCC). CYC_NYCC road condition data is typically used for vehicle simulations and testing to assess the performance of various vehicles under real urban road conditions. One cycle’s total simulation time is 598 s, the waiting time is 210 s, the driving distance is 1.9 km, the maximum speed is 44.58 km/h, the maximum acceleration is 2.68 m/s^2^, and the average acceleration is 0.62 m/s^2^. Figure 4a,b shows the changes in battery SOC and mileage during 80 driving cycles. Overall, it is found that SOC and mileage are approximately linearly correlated with time. That is to say, in the 80 driving cycles, the SOC as a whole is decreasing with time; the mileage shows a continuous increase over time. The total driving time of the car was 47,840 s (13 h 16 min), the driving distance was 1.51278 × 10^5^ m (about 151.3 km), and the remaining battery capacity was 9.53% (about 9.5%). Figure 5a,b shows the required speed of the vehicle under the CYC_NYCC road conditions and the actual speed of the model simulation, from which it can be seen that the results were basically consistent. Figure 5c,d shows the required torque of the vehicle under the CYC_NYCC road conditions and the actual torque obtained from the model simulation. The actual torque was greater during braking, but the results were basically consistent, indicating that the model can meet the requirements of vehicle urban driving. In summary, the Simulink simulation model of the whole vehicle was correct and can meet the requirements of the New York City Road conditions. From the simulation results, the simulation model of the urban mini electric vehicle was considered correct, and the target mileage value was basically consistent with that in the simulation results. These demonstrate that the designed vehicle model can meet the basic requirements of general urban road conditions and personal driving.

## 3. Research on Brushless DC Motor and Its Control Algorithm

### 3.1. Brushless DC Motor Model

A three-phase six-state inductive (Hall sensor) brushless DC motor is considered here [26]. The mathematical model for the brushless DC motor is as follows:

(17) $$u=R_{eq}i+L\frac{di}{dt}+E_{eq}.$$

In particular, Equation (17) is the voltage balance equation, in which ***u*** is the voltage instantaneous value matrix of each phase stator, ***i*** is the current instantaneous value matrix of each phase stator, ***R_eq_*** is the equivalent resistance value matrix of each phase armature, ***L*** is the inductance matrix, and ***E_eq_*** is the instantaneous value matrix of each phase-induced electromotive force.

(18) $$T_{d}=\frac{GD^{2}}{375}\frac{dn}{dt}+T_{l}+B_{N}\frac{n\pi }{30},$$

(19) $$\frac{GD^{2}}{375}=Z.$$

Equation (18) is the mechanical kinetic equation, in which *T_d_* is the electromagnetic torque of the motor, *n* is the actual speed of the motor, *GD*^2^ is the flywheel torque, *T_l_* is the load torque, *B_N_* is the mechanical damping, and ω is the rotor angular velocity. For simplicity, Equation (19) can be substituted into Equation (18). The inductance *L* can be calculated by Equation (20):

(20) $$L=\left[\begin{array}{ccc} L_{aa} & L_{ab} & L_{ac}\\ L_{ba} & L_{bb} & L_{bc}\\ L_{ca} & L_{cb} & L_{cc} \end{array}\right].$$

The brushless DC motor is a three-phase motor, in which the three-phase winding of the three-phase motor is symmetric. In an electric cycle, the mutual inductance between adjacent two phases is the same, and the self-inductance of each phase is the same [27]. This can be expressed by Equations (21) and (22), as follows:

(21) $$L_{ab}=L_{ac}=L_{ba}=L_{bc}=L_{bc}=L_{bc}=M,$$

(22) $$L_{aa}=L_{bb}=L_{cc}=L.$$

(23) $$R_{eq}=\left[\begin{array}{ccc} r & 0 & 0\\ 0 & r & 0\\ 0 & 0 & r \end{array}\right],$$

where *r* is the equivalent resistance of each phase winding. The voltage balance equation can be described by Equation (24):

(24) $$\left[\begin{array}{c} u_{A}\\ u_{B}\\ u_{C} \end{array}\right]=\left[\begin{array}{ccc} r & 0 & 0\\ 0 & r & 0\\ 0 & 0 & r \end{array}\right]\left[\begin{array}{c} i_{A}\\ i_{B}\\ i_{C} \end{array}\right]+\left[\begin{array}{ccc} L & M & M\\ M & L & M\\ M & M & L \end{array}\right]\rho \left[\begin{array}{c} i_{A}\\ i_{B}\\ i_{C} \end{array}\right]+\left[\begin{array}{c} E_{A}\\ E_{B}\\ E_{C} \end{array}\right].$$

where *ρ* denotes that the current of each phase is differentiated from time. The induced electromotive force of each phase winding can be calculated by Equation (25):

(25) $$E_{i}=\sum_{x=1}^{N/2a}B_{x}lv=B_{avg}lv\frac{N}{2a},$$

where *N*/2*a* represents the total number of conductors on a branch, *B_avg_* represents the average magnetic density which can be calculated by Equation (26), *l* is the length of the conductor, and *v* is the speed of the conductor cutting the magnetic induction line, which can be calculated by Equation (27):

(26) $$B_{avg}=\frac{\phi_{m}}{l\tau },$$

(27) $$v=2P\tau \frac{n}{60}.$$

where *ϕ_m_* is the unipolar magnetic flux, and *τ* is the polar distance. *P* is the number of pole-pairs. The expression of induced electromotive force in Equation (28) can be obtained from Equations (25)–(27):

(28) $$E_{i}=C_{e}n.$$

The back electromotive force constant *C_e_* can be calculated by Equation (29):

(29) $$C_{e}=\frac{pN}{60a}\phi_{m}.$$

The electromagnetic torque *T_d_* can be calculated by Equation (30):

(30) $$T_{d}=\sum_{x=1}^{N}T_{x}=T_{avg}N,$$

where *T_avg_* is the average torque of a conductor, which can be calculated by Equation (31):

(31) $$T_{avg}=f_{avg}\frac{D}{2},$$

where *f_avg_* is the average electromagnetic force on a conductor. This can be calculated by Equation (32):

(32) $$f_{avg}=B_{avg}li^{\prime },$$

where *i′* is the current through a single conductor. The electromagnetic torque *T_d_* can also be calculated by Equation (33):

(33) $$T_{d}=C_{d}i,$$

(34) $$C_{d}=\frac{pN}{2a\pi }\phi_{m}.$$

Using the formula above, the torque constant *C_d_* can be obtained. At the same time, *i* can be obtained from Equations (18), (19), and (33) as:

(35) $$i=\frac{Z\frac{dn}{dt}+T_{l}+B_{N}\omega }{C_{d}}.$$

Ignoring the disturbed magnetic field *B_N_* and bringing Equation (35) into the voltage balance equation, we obtain:

(36) $$u=R_{eq}\frac{Z\frac{dn}{dt}+T_{l}}{C_{d}}+L\rho \frac{Z\frac{dn}{dt}+T_{l}}{C_{d}}+C_{e}n.$$

The Laplace transform and deformation can be obtained by Equation (37):

(37) $$N\left(s\right)=\frac{C_{d}}{ZLs^{2}+ZR_{eq}s+C_{d}C_{e}}U\left(s\right)-\frac{Ls+R}{ZLs^{2}+ZR_{eq}s+C_{d}C_{e}}T_{l}\left(s\right).$$

### 3.2. Chaotic Random Grey Wolf Proportional Integral Differential (CR-GWO-PID) Control Algorithm

The Grey Wolf Optimization (GWO) algorithm is a meta-heuristic optimization algorithm that simulates the biological behavior of a grey wolf population [18]. The algorithm is primarily used to simulate and solve the optimization problem through cooperative and competitive biological predation within the grey wolf population. However, the GWO algorithm has the disadvantages of low accuracy, poor global search ability, and falling into local optima. In view of this, a Chaotic Random Grey Wolf Optimization (CR-GWO) algorithm has been developed to address these shortcomings [28]. The essence of the CR-GWO-PID algorithm also lies in its simulation of swarm intelligence through natural behaviors.

(1) Hybrid Optimization of Population Initialization Improvement Strategy Population initialization significantly influences the convergence speed of global optimization. The purpose of initialization is to generate an initial set of solutions within the search space, typically by random means, designed to cover the entire search space. This allows the algorithm to explore a diverse range of potential solutions. At present, Tent, Chebyshev, Gauss, and other chaotic maps are widely used in group optimization algorithms, due to their randomness, repeatability, and non-recombination [29]. On the basis of these advantages, the Kent chaotic map uses a mixed sine and cosine random assignment [30] to initialize the population. The objective is to introduce a degree of randomness into the population initialization process, thereby reducing the risk of convergence to a local optimal. The mathematical model is as follows:

(38) $$x_{k+1}=\{\begin{array}{c} \frac{x_{k}}{\mu }\;0<x_{k}\leq \mu \\ \frac{1-x_{k}}{1-\mu }\;\mu <x_{k}<1 \end{array},$$

(39) $$x_{k+1}=\left[u\times \cos\left(p\times \pi \right)\times \sin\left(v\times \pi \right)\right]\times x_{k}.$$

**Figure 6:**
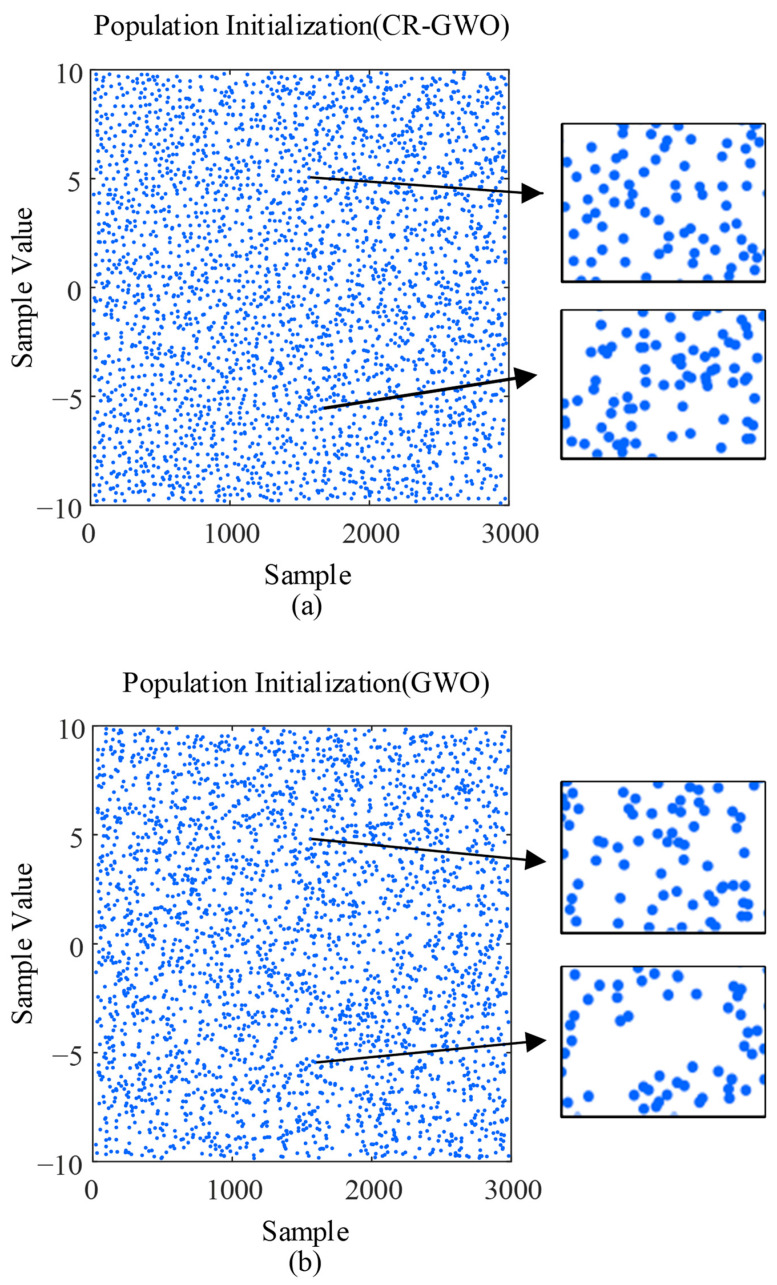
Algorithm population initialization comparison diagram: (**a**) and (**b**).

In order to avoid falling into a short period during initialization, it is necessary to ensure that the initial value is not equal to the control parameter *μ* in Equation (38); furthermore, *μ* ≠ 0.5 and *μ* ∈ (0, 1). At the same time, in order to avoid the emergence of repeated populations, a strategy of sine and cosine random allocation can be adopted on the basis of the Kent chaotic map. In Equation (39), the weight coefficient *u* = 2, the random coefficient *p* ∈ [0, 1], and the random coefficient *v* ∈ [0, 1]. Then, *x_k_* is the chaotic sequence value obtained in the *k*th iteration. As shown in Figure 6, first, the region of the sampled value was divided into 10 × 10 grids (the grid is the calculation method program running is the simulation step) to calculate the number of midpoints of each unit grid, following which the density distribution value was calculated by kernel density estimation (KDE). The density distribution value for the CR-GWO algorithm was 0.020737, while the density distribution value for the GWO algorithm was 0.0015117. Therefore, compared with the GWO algorithm, the CR-GWO algorithm had a more uniform distribution, higher randomness, and less repeatability.

**Figure 7:**
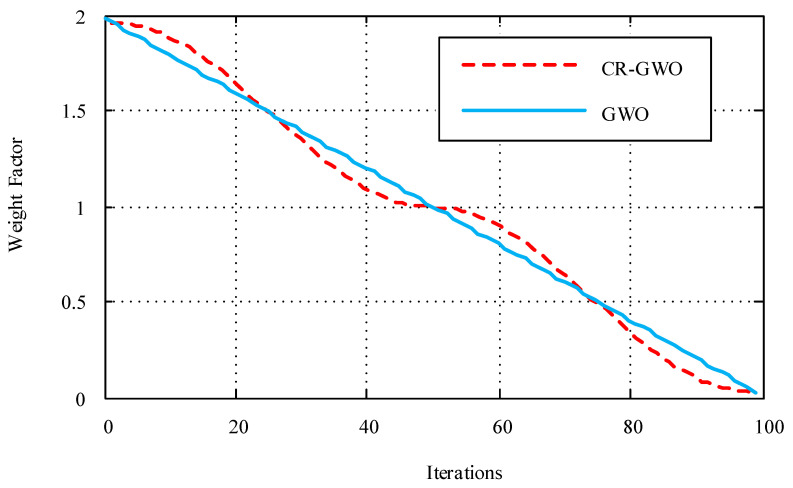
Algorithm distance weight comparison diagram.

(2) Non-Linear Inertia Decreasing Improvement Strategy with Weight Factors. When searching for prey, wolves are affected by a distance weight *A*. When |*A*| > 1, the grey wolf is far away from the prey, and a large-scale search allows for finding the optimal solution in a more global manner. When |*A*| < 1, the search range shrinks and the pursuit of the prey begins [31]. Compared with the linear decreasing strategy in the GWO algorithm, the improved CR-GWO algorithm introduces a sinusoidal non-linear decreasing factor, which enhances the global search ability in the early stage, improves the local search ability in the later stage, and improves the accuracy of the optimal solution of the algorithm [32]. At the same time, it helps the algorithm explore a wide range of solution Spaces and increases the possibility of finding promising regions. The expression of the sinusoidal non-linear decreasing factor is:

(40) $$p=\{\begin{array}{c} 2-{\left(\sin(\frac{\pi (t-1)}{t_{\max}-1})\right)}^{n}\;0<t<\frac{2}{t_{\max}}\\ {\left(\sin(\frac{\pi (t-1)}{t_{\max}-1})\right)}^{n}\;\frac{2}{t_{\max}}\leq t\leq t_{\max} \end{array}.$$

where *t* is the current number of iterations, *t_max_* is the maximum number of iterations and *n* is the decreasing weight; *n =* 2 was selected for this study and the maximum number of iterations was set to 100. The GWO algorithm was compared with the CR-GWO algorithm, and the results are shown in Figure 7.

**Figure 8:**
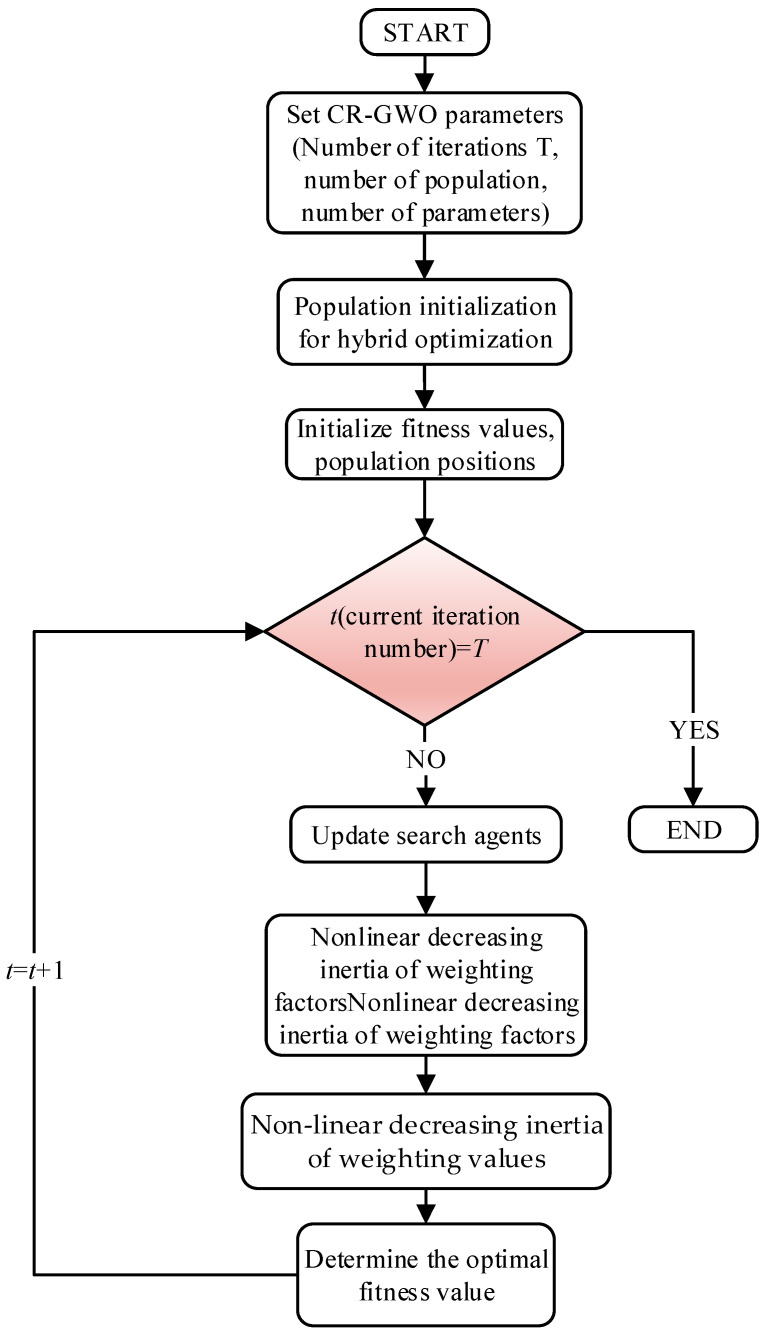
Algorithm flowchart.

(3) Random Proportional Displacement Strategy. A random proportional weight was added to the CR-GWO algorithm [33]. By introducing randomness within the population, the algorithm can converge more swiftly toward potential solutions, reducing unnecessary iteration cycles during the search process and thereby enhancing convergence speed. Simultaneously, the random proportional displacement strategy somewhat reduces the positional disparities among individuals within the population. This aids in accelerating the local search process, enabling the algorithm to converge more rapidly toward the vicinity of local optima, thereby enhancing the algorithm’s precision in local search. The mathematical model expression is:

(41) $$m_{1}=\frac{\left|X_{1}\right|}{\left|X_{1}\right|+\left|X_{2}\right|+\left|X_{3}\right|},$$

(42) $$m_{2}=\frac{\left|X_{2}\right|}{\left|X_{1}\right|+\left|X_{2}\right|+\left|X_{3}\right|},$$

(43) $$m_{3}=\frac{\left|X_{3}\right|}{\left|X_{1}\right|+\left|X_{2}\right|+\left|X_{3}\right|},$$

(44) $$X\left(t+1\right)=\frac{m_{1}\times X_{1}+{2\times \rho \times m}_{2}\times X_{2}+\rho \times m_{3}\times X_{3}}{3}.$$

when the mathematical model is abstracted as a plane image, *X*_1_, *X*_2_, and *X*_3_ are the coordinates of the three vertices of the triangle, where *m*_1_, *m*_2_, and *m*_3_ represent the weight of the distance ratio at any point within the triangle. *X*_1_, *X*_2_, and *X*_3_ correspond to three grey wolves, denoted alpha, beta, and delta, respectively. They determine the location of omega wolves that are constantly close to their prey for attack. The random expansion of the weight factor *ρ* ∈ (0, 1) expands the distance ratio of *X*_2_ and *X*_3_ to the internal point, which means that the internal point is closer to *X*_1_. In the algorithm, the position of omega is closer to that of alpha, and alpha’s position is closer to the prey, such that the algorithm needs fewer iterations when seeking the optimal solution and the convergence speed is faster. Based on this improvement, the implementation steps for the CR-GWO algorithm are shown in Figure 8.

In order to verify its optimization ability six test functions (three unimodal benchmark functions and three multimodal benchmark functions) were selected to test the global solution ability of CR-GWO algorithm and the ability to jump out of local optima, in comparison with the GWO algorithm. The test functions are detailed in Table 2.

**Figure 9:**
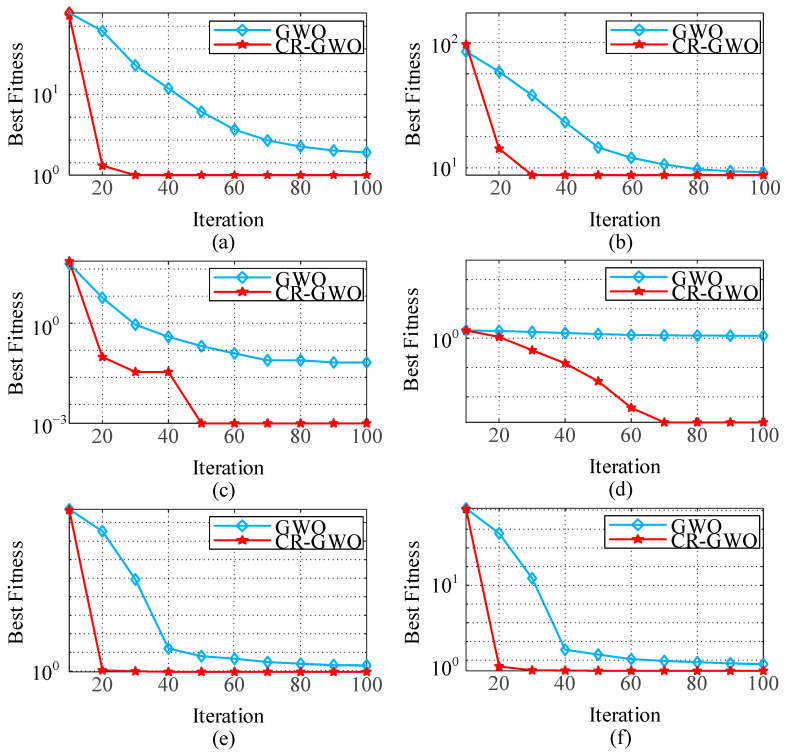
Test function comparison results: (**a**) Test function *f*_1_; (**b**) test function *f*_2_; (**c**) test function *f*_3_; (**d**) test function *f*_4_; (**e**) test function *f*_5_; and (**f**) test function *f*_6_.

The number of populations was set to 50 and the maximum number of iterations was 100. In order to fully verify the convergence accuracy and evaluation stability of the proposed algorithm to avoid randomness, the CR-GWO and GWO were each run 50 times, and the average and standard deviation of the optimal fitness were taken to measure the performance of the algorithms. The results and data are shown in Figure 9.

The data results after 50 runs are provided in Table 3. Compared with the GWO algorithm, the solution accuracy of the CR-GWO algorithm was improved by about 25.13%, 13.68%, 32.65%, 39.20%, 30.82%, and 34.46% on the six test functions, respectively, with an average increase of about 29.323%. In addition, compared with the GWO algorithm, the CR-GWO algorithm consistently obtained the optimal solution with fewer iterations. These results demonstrate that the CR-GWO algorithm has faster convergence speed, higher convergence accuracy, and stronger stability.

**Figure 10:**
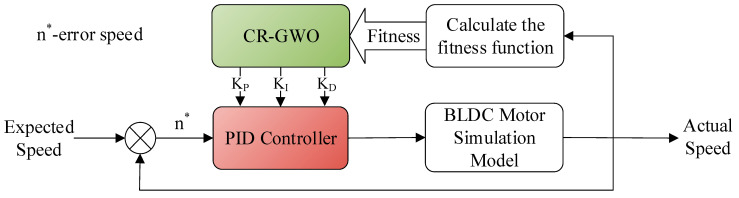
CR-GWO-PID motor speed simulation flow diagram.

**Figure 11:**
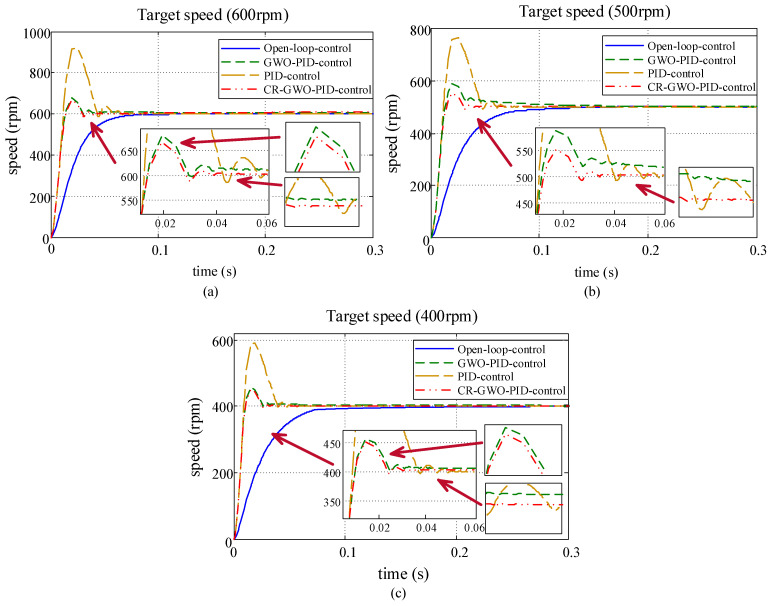
Motor speed simulation curves under the four control strategies: (**a**) Target speed 600 rpm; (**b**) target speed 500 rpm; and (**c**) target speed 400 rpm. (The red arrow indicates magnification of the specified location.).

On this basis, the parameters for the PID controller of the in-wheel motor were adjusted using the CR-GWO optimization algorithm, and a motor simulation experiment was carried out using the optimized parameters [34,35]. The parameters for the brushless DC motor are shown in Table 4. The preset range of PID parameters was [0, 10], the number of iterations of the algorithm was 100, and the number of populations was 30. In order to test the superiority of the improved CR-GWO-PID control algorithm, compared with other control strategies, open-loop control, self-designed PID parameter value control (PID parameters tuned based on engineering experience), and GWO-PID control were compared in the test. The test was carried out at different speeds (600 rpm, 500 rpm, and 400 rpm) [25]. The motor simulation process is shown in Figure 10, and the self-designed PID parameter values were *K_P_* = 0.5, *K_I_* = 0.005, and *K_D_* = 0.001 [36,37]. The effects of the four different control methods are shown in Figure 11.

According to Table 5, Table 6 and Table 7, CR-GWO-PID control demonstrated significant advantages over the other control methods in the simulations at three different speeds. Specifically, when compared with the open-loop control, the rise time of the CR-GWO-PID control was reduced by about 41.25% on average, while the settling time was reduced by 41.92% on average; compared with the self-designed PID control, the rise time of the CR-GWO-PID control was reduced by about 1.587% on average, the settling time was reduced by 20.97% on average, and the peak time was reduced by 27.96% on average. Compared with the GWO-PID control, the rise time of the CR-GWO-PID control was reduced by about 0.957% on average, the settling time was reduced by 9.32% on average, and the peak time was reduced by 1.90% on average. In addition, during simulations at three different speeds, the overshoot of the CR-GWO-PID control did not exceed the range of 10–20%, indicating excellent control accuracy. Open-loop control does not have an effective control effect. While the self-designed PID control exhibits good acceleration performance, it suffers from excessive overshoot. Additionally, manually tuning PID parameters requires extensive testing to achieve the desired results. Compared to the optimized algorithm-calculated PID parameters, it shows significant disadvantages and is therefore not suitable. In contrast to the first two control methods, under GWO-PID control, the system shows relatively good results in terms of overshoot and settling time calculation. However, under CR-GWO-PID control, the system can achieve better acceleration performance and stability with lower overshoot.

The results above demonstrate that the CR-GWO-PID control algorithm presents excellent dynamic performance in the case of simulating different speeds, with a faster rise time, shorter settling time, and lower overshoot, while maintaining excellent acceleration performance and stability.

## 4. Bench Test of In-Wheel Motor Control System

### 4.1. Construction of Motor Test Bench

The MCU for actual motor speed acquisition and motor control was composed of electronic components including an ARM processor (model GD32F103VCT6), analog switch IC (DG408DY), and drive signal IC (LJ245A). The MCU also incorporates peripheral functions such as PWM signal output and SDIO protocol support. For the test, a brushless DC motor with a three-phase bridge rectifier circuit (output 48 V control voltage), a sensor (Hall sensor), and a magnetic powder brake (model EL-PB-5; to provide a load for the drive motor) were used. At the same time, a speed sensor (model DYN-200) was used to measure the speed of the motor in the bench test.

**Figure 12:**
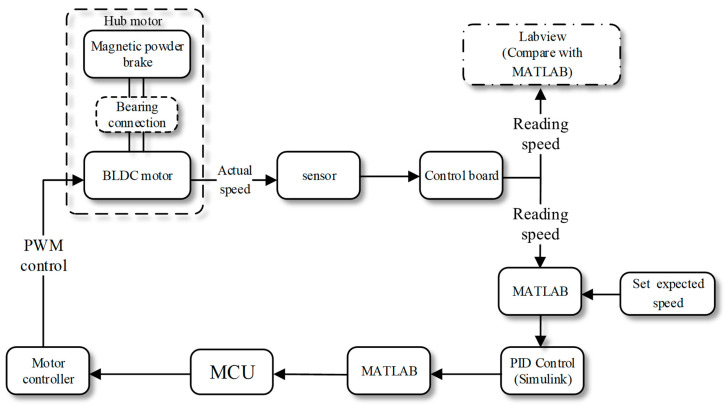
Flowchart of the in-wheel motor test. (The solid line represents the process and the dashed line represents the constituent structure.).

**Figure 13:**
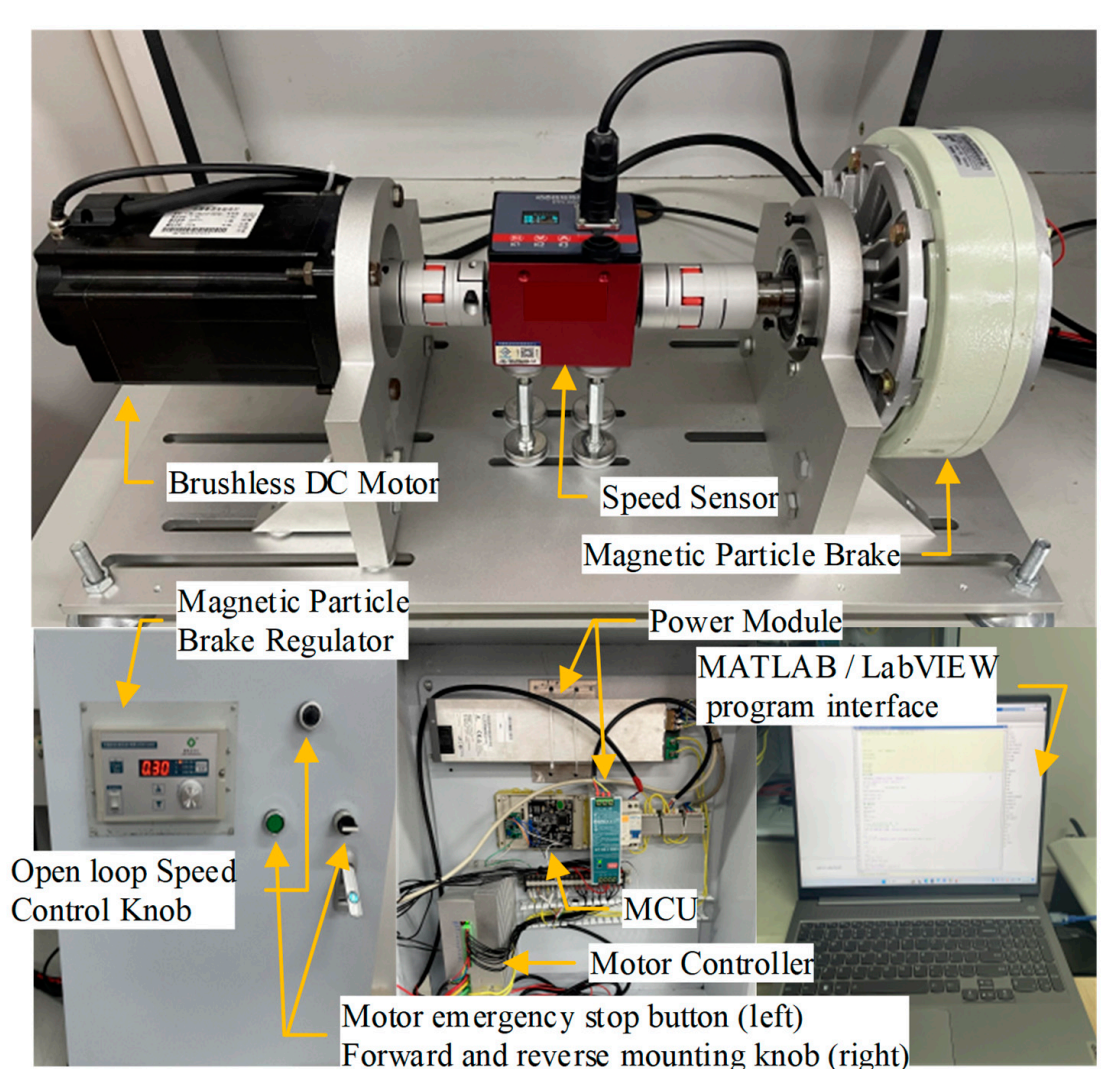
Images of the test bench, showing its layout.

The bench test process is illustrated in Figure 12. During the whole experiment, the brushless DC motor and the magnetic powder brake simulated the in-wheel motor drive system. The DYN-200 sensor collected the speed signal and transmitted it to the MCU. A program written using MATLAB software was used to read the speed signal and preset the expected speed, then control the motor speed through the PID controller optimized using the CR-GWO-PID control algorithm. At the same time, the MATLAB program also outputs control instructions, such that the MCU could output the corresponding PWM signal to the motor controller, thus realizing a series of acquisition, optimization, and control operations. An image of the physical test bench is shown in Figure 13.

### 4.2. Test Results and Analysis

The analysis of the results from the motor simulation indicates that the CR-GWO-PID control algorithm exhibits excellent control performance. Consequently, to validate the real-world performance of the in-wheel motor test bench based on the CR-GWO-PID control algorithm, bench tests were conducted at speeds of 400 rpm, 500 rpm, and 600 rpm, respectively.

**Figure 14:**
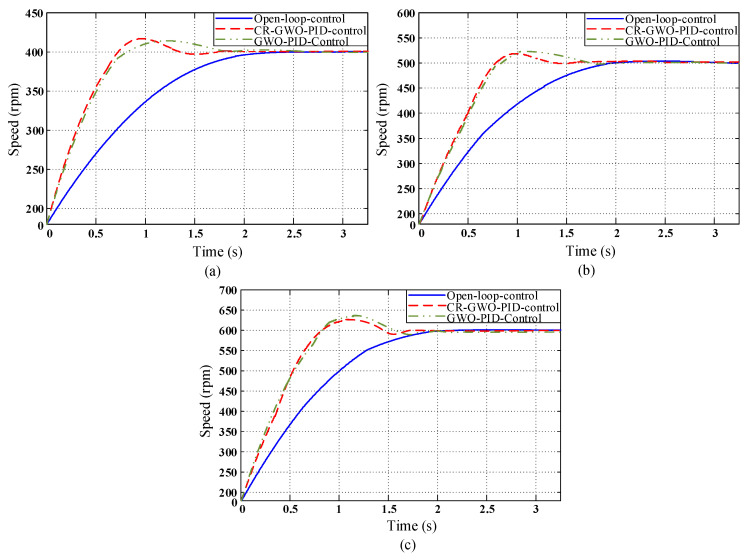
In Test Group 1, the Simulation curve diagram of motor speed under the four control strategies: (**a**) Target speed 400 rpm; (**b**) target speed 500 rpm; and (**c**) target speed 600 rpm.

**Figure 15:**
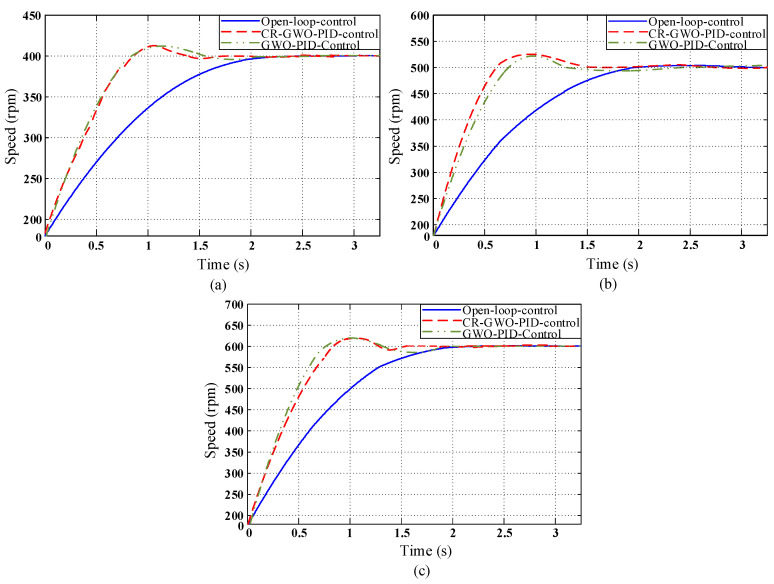
In Test Group 2, the Simulation curve diagram of motor speed under the four control strategies: (**a**) Target speed 400 rpm; (**b**) target speed 500 rpm; and (**c**) target speed 600 rpm.

**Figure 16:**
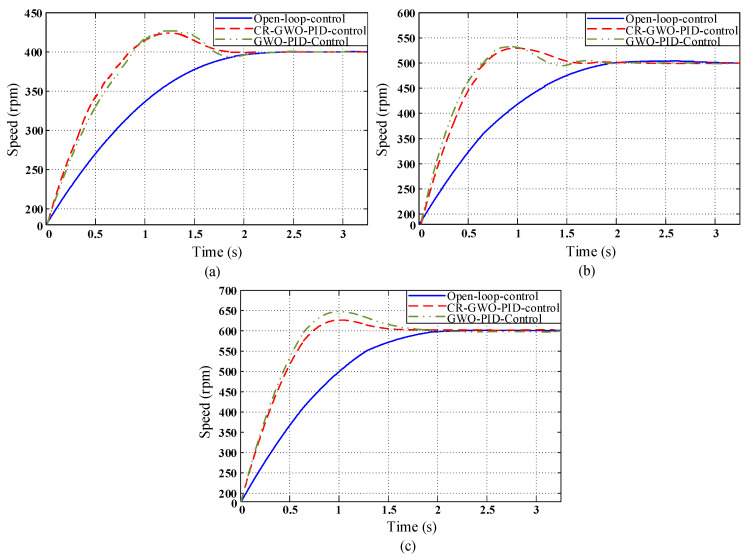
In Test Group 3, the Simulation curve diagram of motor speed under the four control strategies: (**a**) Target speed 400 rpm; (**b**) target speed 500 rpm; and (**c**) target speed 600 rpm.

The PID parameters, as shown in Table 8, Table 9 and Table 10, were calculated for motor simulation at different speeds. However, it is necessary to select the optimal parameters for the PID controller to meet the requirements of different speeds. Therefore, tests were conducted at different speeds for each set of PID parameters., and the test results are depicted in Figure 14, Figure 15 and Figure 16.

In the PID parameter testing conducted in Test Group 1, as indicated by Table 11, Table 12 and Table 13, under the three different speed test conditions, when the CR-GWO-PID control was compared with open-loop control, the rise time was shortened by 29.04% on average, while the settling time was shortened by 35.47% on average; compared with the GWO-PID control, the rise time was shortened by 5.41% on average, and the settling time was shortened by 14.72% on average. The peak time was shortened by 9.63% on average. In addition, the overshoot of CR-GWO-PID control was stable within 5% under the three working conditions.

In the PID parameter testing conducted in Test Group 2, as indicated by Table 14, Table 15 and Table 16, under the three different speed test conditions, when the CR-GWO-PID control was compared with open-loop control, the rise time was shortened by 33.02% on average, while the settling time was shortened by 29.97% on average; compared with the GWO-PID control, the rise time was shortened by 3.92% on average, and the settling time was shortened by 24.81% on average. The peak time was shortened by 6.07% on average. In addition, the overshoot of CR-GWO-PID control was stable within 5% under the three working conditions.

In the PID parameter testing conducted in Test Group 3, as indicated by Table 17, Table 18 and Table 19, under the three different speed test conditions, when the CR-GWO-PID control was compared with open-loop control, the rise time was shortened by 32.45% on average, while the settling time was shortened by 23.86% on average; compared with the GWO-PID control, the rise time has remained nearly unchanged, and the settling time was shortened by 17.32% on average. The peak time was shortened by 0.85% on average. In addition, the overshoot of CR-GWO-PID control was stable within 5% under the three working conditions.

Based on the comparison of data from the three bench test groups, several key conclusions can be drawn. Firstly, it is evident that the performance of the motor under the CR-GWO-PID control algorithm surpasses that of alternative control methods. Secondly, recognizing the paramount importance of stability and safety during vehicle operation, settling time has been selected as a pivotal metric. In Test Group 2, when computed using the CR-GWO-PID control algorithm, the motor exhibits a significantly reduced settling time, aligning more closely with the final parameters set by the PID controller. These collective results underscore the substantial advantages of the CR-GWO-PID control algorithm for in-wheel motor-driven electric vehicles, effectively enhancing both the maneuverability and overall safety of the vehicles during linear motion.

## 5. Conclusions

In order to improve the performance of micro-electric vehicles, this paper studied the speed control systems of four-wheel drive micro-electric vehicles driven by in-wheel motors. The following conclusions were drawn: (i) The simulation results demonstrated that the studied micro-electric vehicle model is reasonable and feasible, allowing for road condition simulations to obtain comprehensive motor data. The driving range of the micro-electric vehicle reached about 151.3 km and the remaining power was about 9.5%. Under general driving conditions, it suits the purpose of urban transportation; (ii) the simulation results obtained for the six test functions indicated that through the use of the three different improvement strategies proposed in this paper—namely, integrating a population initialization approach using sine and cosine random distribution factors in the Kent chaotic map, algorithm weighting factors that decrease non-linearly through the use of a sine function, and movement of the population position in a random proportional manner—the global optimization ability, convergence speed, and optimization accuracy of the traditional grey wolf algorithm can be improved; and (iii) Simulation and bench test results demonstrate that the application of the CR-GWO algorithm to the motor PID control system improves the speed control effectiveness of the drive motor. Comprehensive bench test data reveals that the motor, when controlled by the CR-GWO-PID algorithm, exhibits reduced rise time, settling time, and peak time compared to other control methods. This results in an overall enhancement of system performance. The development of this control algorithm, coupled with the fine-tuning of PID parameters, enhances motor stability across various speed ranges, consequently improving the safety and maneuverability of micro-electric vehicles equipped with four-wheel in-wheel motor drive systems.

## Figures and Tables

**Table 1:** Vehicle parameter table.

Vehicle Parameters	Values
vehicle mass	665 kg
Length × width × height	2920 mm × 1493 mm × 1621 mm
Wheelbase	1940 mm
Wheel pitch	1290 mm
Minimum ground clearance	125 mm
Rolling damping coefficient	0.015
Air drag coefficient	0.35
Tire radius	385 mm
Area of the windward zone	2.178 m^2^

**Table 2:** Description of benchmark functions.

Function	Dim	Range	$f_{min}$
$f_{1}=\sum_{i=1}^{n-1}\left[100{\left(x_{i+1}-x_{i}{}^{2}\right)}^{2}+{\left(x_{i}-1\right)}^{2}\right]$	30	[−30, 30]	0
$f_{2}=\sum_{i=1}^{n}\left[{\left(x_{i}+0.5\right)}^{2}\right]$	30	[−100, 100]	0
$f_{3}=\sum_{i=1}^{n}\left(ix_{i}^{4})+random[0,1\right]$	30	[−1.28, 1.28]	0
$f_{4}=-20\exp(-0.2\sqrt{\frac{1}{n}\sum_{i=1}^{n}\left(x_{i}^{2}\right)})-\exp(\frac{1}{n}\sum_{i=1}^{n}\left(\cos(2\pi x_{i})\right))+20+e$	30	[−5.12, 5.12]	0
$f_{5}=\frac{1}{4000}\sum_{i=1}^{n}\left(x_{i}^{2}\right)-\prod_{i=1}^{n}\left(\cos(\frac{x_{i}}{\sqrt{i}})\right)+1$	30	[−600, 600]	0
$\begin{array}{l} f_{6}=\frac{\pi }{n}\{10\sin(\pi y_{{}_{1}})+{\sum_{i=1}^{n-1}\left(y_{{}_{i}}-1\right)}^{2}[10\sin^{2}(\pi y_{i+1})]+{\left(y_{n}-1\right)}^{2}\}\\ \;+\sum_{i+1}^{n}u(x_{i},10,100,4)\\ \begin{array}{l} y_{i}=1+\frac{x_{i}+1}{4}\\ u(x_{i},10,100,4)=\left\{\begin{array}{cc} k{\left(x_{i}-a\right)}^{m} & x_{i}>a\\ 0 & -a<x_{i}<a\\ k{\left(-x_{i}-a\right)}^{m} & x_{i}<-a \end{array}\right. \end{array} \end{array}$	30	[−50, 50]	0

**Table 3:** Analysis of test results.

Function	GWO		CR-GWO	
	AVG	STD	AVG	STD
$f_{1}$	1.2971927	0.623971	0.97100573	0.12340483
$f_{2}$	5.83278437	0.402126723	5.034828256	0.320877835
$f_{3}$	0.002948175	0.025320668	0.001975758	0.001219342
$f_{4}$	1.0237778202	1.8486 × 10^−10^	0.623297658	1.70004 × 10^−15^
$f_{5}$	1.388772273	1.347839087	0.935870771	0.163039133
$f_{6}$	1.482505429	2.815509853	0.972366285	0.046705182

**Table 4:** Brushless DC motor parameters.

BLDC Motor Parameters	Values
BLDC Motor rating	48 V, 2000 W
Rated speed	3000 rpm
Rated torque	6.4 N·m
Moment of inertia	14.6 × 10^−4^ kg·m^2^
Weight	7 kg
Torque constant	0.123 N·m/A
Armature resistance	0.4605 Ω
Armature inductance	3.226 mH

**Table 5:** The 600 rpm simulation results. (Rise Time: The time it takes for the signal to transition from the initial value of 10% to the first time it reaches the stable value of 90%. Overshoot: The magnitude by which a signal exceeds its stable value for the first time, typically near the beginning of its response. Settling Time: The duration required for a signal, after overshooting, to ultimately stabilize near its stable value. Peak Time: The time taken for a signal to reach its maximum overshoot value. “\” indicates ellipsis.).

Algorithm	Rise Time (s)	Overshoot (%)	Settling Time (s)	Peak Time (s)
Open-loop	0.0898	0	0.153	\
PID	0.0196	47.75	0.104	0.0231
GWO-PID	0.0192	14.075	0.0539	0.0199
CR-GWO-PID	0.0189	12.5	0.0521	0.019

**Table 6:** The 500 rpm simulation results.

Algorithm	Rise Time (s)	Overshoot (%)	Settling Time (s)	Peak Time (s)
Open-loop	0.0832	0	0.2153	\
PID	0.0195	53.10	0.0829	0.0293
GWO-PID	0.019	17.68	0.2252	0.0191
CR-GWO-PID	0.0193	10.40	0.06492	0.0192

**Table 7:** The 400 rpm simulation results.

Algorithm	Rise Time (s)	Overshoot (%)	Settling Time (s)	Peak Time (s)
Open-loop	0.0743	0	0.2736	\
PID	0.0195	53.23	0.1126	0.0362
GWO-PID	0.0191	12.717	0.0712	0.0187
CR-GWO-PID	0.0192	11.133	0.0548	0.0175

**Table 8:** PID parameter table for 400 rpm bench test motor (Test Group 1).

Algorithm	*K_P_*	*K_I_*	*K_D_*
GWO-PID	1.035	0.3565	0.0029605
CR-GWO-PID	1.01	0.38985	0.002655

**Table 9:** PID parameter table for 500 rpm bench test motor (Test Group 2).

Algorithm	*K_P_*	*K_I_*	*K_D_*
GWO-PID	1.016	0.41862	0.00235
CR-GWO-PID	1.105	0.41985	0.0029605

**Table 10:** PID parameter table for 600 rpm bench test motor (Test Group 3).

Algorithm	*K_P_*	*K_I_*	*K_D_*
GWO-PID	1.308	0.3497	0.002089
CR-GWO-PID	1.017	0.3565	0.0029605

**Table 11:** Test Group 1, the 400 rpm bench test data sheet.

Algorithm	Rise Time (s)	Overshoot (%)	Settling Time (s)	Peak Time (s)
Open-loop	1.25	0	2.45	\
GWO-PID	0.808	3.623	1.825	1.46
CR-GWO-PID	0.794	3.775	1.57	0.98

**Table 12:** Test Group 1, the 500 rpm bench test data sheet.

Algorithm	Rise Time (s)	Overshoot (%)	Settling Time (s)	Peak Time (s)
Open-loop	1.22	0	2.15	\
GWO-PID	0.973	3.525	1.986	1.217
CR-GWO-PID	0.897	3.429	1.495	0.996

**Table 13:** Test Group 1, the 600 rpm bench test data sheet.

Algorithm	Rise Time (s)	Overshoot (%)	Settling Time (s)	Peak Time (s)
Open-loop	1.08	0	1.98	\
GWO-PID	0.813	7.5	1.886	1.314
CR-GWO-PID	0.819	4.723	1.685	1.209

**Table 14:** Test Group 2, the 400 rpm bench test data sheet.

Algorithm	Rise Time (s)	Overshoot (%)	Settling Time (s)	Peak Time (s)
Open-loop	1.25	0	2.45	\
GWO-PID	0.796	3.04	1.825	1.364
CR-GWO-PID	0.802	3.09	1.57	1.08

**Table 15:** Test Group 2, the 500 rpm bench test data sheet.

Algorithm	Rise Time (s)	Overshoot (%)	Settling Time (s)	Peak Time (s)
Open-loop	1.22	0	2.15	\
GWO-PID	0.85	4.125	2.06	0.95
CR-GWO-PID	0.75	4.529	1.49	0.97

**Table 16:** Test Group 2, the 600 rpm bench test data sheet.

Algorithm	Rise Time (s)	Overshoot (%)	Settling Time (s)	Peak Time (s)
Open-loop	1.08	0	1.98	\
GWO-PID	0.725	3.05	2.26	1.06

**Table 17:** Test Group 3, the 400 rpm bench test data sheet.

Algorithm	Rise Time (s)	Overshoot (%)	Settling Time (s)	Peak Time (s)
Open-loop	1.25	0	2.45	\
GWO-PID	0.807	4.043	2.025	1.289
CR-GWO-PID	0.803	4.05	1.803	1.256

**Table 18:** Test Group 3, the 500 rpm bench test data sheet.

Algorithm	Rise Time (s)	Overshoot (%)	Settling Time (s)	Peak Time (s)
Open-loop	1.22	0	2.15	\
GWO-PID	0.736	4.125	1.832	0.98
CR-GWO-PID	0.796	4.029	1.69	1.03

**Table 19:** Test Group 3, the 600 rpm bench test data sheet.

Algorithm	Rise Time (s)	Overshoot (%)	Settling Time (s)	Peak Time (s)
Open-loop	1.08	0	1.98	\
GWO-PID	0.73	7.333	2.26	1.05
CR-GWO-PID	0.79	3.167	1.509	1.02

## Data Availability

Not applicable.
